# Urban-rural differences in the impacts of multiple chronic disease on functional limitations and work productivity among Chinese adults

**DOI:** 10.1080/16549716.2021.1975921

**Published:** 2021-09-17

**Authors:** Yang Zhao, Li He, Chunlei Han, Brian Oldenburg, Grace Sum, Tilahun Nigatu Haregu, Xiaoyun Liu

**Affiliations:** a The George Institute for Global Health at Peking University Health Science Center, Beijing, China; b The George Institute for Global Health, University of New South Wales, Sydney, New South Wales, Australia; c College of Physical Education and Sport, Beijing Normal University, Beijing, China; d College of Public Health and Management, Binzhou Medical University, Yantai, Shandong 264003, China; e School of Public Health and Preventive Medicine, Monash University, Melbourne, Victoria, Australia; f Baker Heart and Diabetes Institute, Melbourne, Victoria, Australia; g Academic Research Collaboration in Health, Alfred Hospital, Melbourne, Victoria, Australia; h Saw Swee Hock School of Public Health, National University of Singapore, Singapore; i China Centre for Health Development Studies, Peking University, Beijing, China

**Keywords:** Multimorbidity, functional limitations, work productivity, china

## Abstract

**Background:**

Chronic disease multimorbidity has become a major challenge for health systems. While a lot of research has evaluated the direct economic burden of multimorbidity on health care utilization and cost, little attention has been given to the impacts on work productivity and functional limitations, as indirect indicators of disease burden.

**Objectives:**

This study aims to examine the prevalence of multimorbidity among Chinese adults and its impact on functional disability and work productivity. It also investigates urban-rural differences in these relationships.

**Method:**

This study utilized the data from the China Health and Retirement Longitudinal Study (CHARLS) in 2015, including 11,176 participants aged 45 years and older. Multivariable logistic regression models were used to estimate the effect of multimorbidity on functional disability (i.e. ADL: activities of daily life; IADL: instrumental activities of daily life), and work productivity loss due to health problems. Negative binomial regression models were used to assess the association of multimorbidity with sickness absences from agricultural work and employed non-agricultural work.

**Results:**

68.8% of total participants in CHARLS had multimorbidity in China in 2015. Rural residents with multimorbidity reported higher proportions of physical functions and days of sick leave than urban residents. Multimorbidity was positively associated with ADL limitation (odds ratio 1.924, 95% CI 1.656–2.236), IADL limitation (1.522, 1.326–1.748), limited work due to health problems (1.868, 1.601–2.178) and days of sick leave (for agricultural work, incidence rate ratio 1.676, 95% CI 1.390–2.020; for employed non-agricultural work, 2.418, 1.245–4.696). For the rural group, the impact of multimorbidity on functional limitations and work productivity loss (except for early retirement), was less than the urban group.

**Conclusions:**

Multimorbidity poses significant challenges for functional health and work productivity These have significant negative economic consequences for individuals, the Chinese health system and the society.

## Background

Chronic diseases are a leading cause of global morbidity and mortality, with over 80% of chronic non-communicable disease (NCD) mortality occurring in low-and middle-income countries (LMICs), such as China [1,2]. The ageing population, coupled with the increasing prevalence of NCD risk factors, have led to a rise in the prevalence of multimorbidity. Multimorbidity is defined as an individual with two or more co-existing physical or mental health conditions. Recent studies in China have found that even though the overall prevalence of multimorbidity is estimated to be 61% in Chinese adults, the prevalence of multimorbidity is even higher (71%)for those aged 75 years and above [3,4]. With the rapidly increasing age of the Chinese population and the increasing levels of multimorbidity, it is imperative to understand the impacts of multimorbidity on individuals’ daily life and the broader society as this is crucial for diminishing the burden of chronic diseases in China.

An important impact of the individual burden of disease is the decline in individuals’ functional capacities in activities of daily life. Additionally, there is the direct economic burden of increased health care costs as well as the indirect economic burden that includes productivity loss at work. While health care utilization and the increased costs for people with multimorbidity in China and other countries have been widely evaluated [5–12], little research has evaluated the impacts on the functional capacity and work productivity of Chinese adults and whether there are differences in these impacts between rural and urban populations in China. Although significant relationships between multimorbidity and increased difficulties in activities and instrumental activities of daily life have been observed in Chinese adults [13–15], the indirect economic burden of multimorbidity related to productivity loss at work in China is still relatively unknown.

A few studies have investigated the impact of multimorbidity on work productivity in high-income countries, such as the USA, Netherlands, Australia, and Japan [16–21]. Several outcomes related to work productivity have been assessed, including the incidence and duration of absenteeism, risk of long-term absenteeism (six or more consecutive weeks), unemployment (labor force participation), early retirement, presenteeism and critical incidents [21]. But the conclusions and generalizability of these studies were limited by their small sample sizes and these studies have mostly been undertaken in high income countries, rather than in large and rapidly changing middle income countries like China [19,21]. In a study examining the association between physical conditions and non-agricultural work productivity loss among the middle-aged Chinese population, Pan and colleagues examined the association between mental-physical multimorbidity and disability, and work productivity [13]. However, the urban-rural difference in relation to physical conditions and work productivity has not been investigated in the Chinese population [13,22].

It well known that there are great disparities in socioeconomic status, quality of life, and health services utilization between urban and rural residents in China [23–26]. For example, elderly rural people are less likely to use inpatient services than those living in urban areas [23]. Also, several previous studies have identified that residence (i.e. rural and urban) are important influential factors which are related to living with a disability [27,28]. Further, the difference in the prevalence of both single chronic diseases and multimorbidity has been observed between rural and urban populations [29–31]. However, no study has investigated whether the associations between multimorbidity and functional limitations and productivity loss vary between rural and urban populations in China.

To address this knowledge gap, this study uses population-level and nationally-representative data from China, the most populous country and the second-largest economy in the world, in order to examine the rural-urban differences in associations between physical multimorbidity and functional limitations and work productivity loss among Chinese adults.

## Methods

### Data sources

This study used the second follow-up wave (2015) of data from the China Health and Retirement Longitudinal Study (CHARLS). The study collected high-quality data from a nationally representative sample of Chinese residents aged 45 and older, using multi-stage stratified probability-proportionate-to-size sampling. We used the second follow-up wave of CHARLS 2015 because it used objective measures including blood pressures, HbA1c, triglycerides, and total cholesterol, etc. Such measures are important and essential in order to be able to identify people with hypertension, diabetes and dyslipidemia more accurately and reliably than the most recent wave (2018) of CHARLS which only contained self-reported indicators.

The total sample size of the CHARLS baseline survey was 17,708 individual respondents. Ongoing follow-up surveys were conducted once every two or three years. A detailed description of the survey objectives and methods has been reported elsewhere [32]. For this study, we identified 14,576 respondents without loss to follow-up. After removing respondents aged below 45 years and individuals with missing values of dependent or independent variables, our final sample had 11,176 respondents (76.7% of 14,576). Functional limitations were analysed using this entire sample, and sick leave days for employed non-agricultural work was analysed among the working-age population (5, 971 respondents) in China, defined as those who were aged below 55 years for females and 60 years for males.

### Measures

In this study, multimorbidity was defined as the presence of two or more chronic conditions [33,34]. A total of 12 chronic diseases were used to measure physical multimorbidity, including hypertension, diabetes and dyslipidaemia which were measured based on biomarkers or blood test information, and 9 self-reported diagnosed chronic diseases (heart disease, stroke, cancer, chronic lung disease, digestive disease, liver disease, kidney disease, arthritis, and asthma). We counted the number of chronic diseases for each participant to identify those with multimorbidity.

In CHARLS, each respondent’s systolic blood pressure (SBP) and diastolic blood pressure (DBP) were recorded three times by a trained nurse using a HEM-7112 electronic monitor (Omron, Kyoto, Japan). The mean values for each respondent were then calculated but only given to the respondents once the interviews had ended. Diagnosed hypertension was defined as SBP ≥140 mmHg and/or DBP ≥90 mmHg by calculating the three mean readings, and/or being on antihypertensive medication for raised blood pressure [35,36]. In this study, diabetes was defined by 1) a fasting plasma glucose level of ≥126 mg/dL (7.0 mmol/L); and/or 2) HbA1c concentration of ≥6.5%; and/or 3) being insulin treatment and/or taking medication for raised blood sugar [37,38]. Dyslipidaemia was defined by 1) total cholesterol (TC) ≥ 240 mg/dL (6.22 mmol/L); and/or 2) low-density lipoprotein cholesterol (LDL-C) ≥ 160 mg/dL (4.14 mmol/L); and/or 2) high-density lipoprotein cholesterol (HDL-C) <40 mg/dL (1.04 mmol/L); and/or 2) triglyceride (TG) ≥ 200 mg/dL (2.26 mmol/L); and/or 2) taking anti-dyslipidaemia medication [39,40].

For primary outcomes of interest, the activities of daily living (ADL) and the instrumental activities of daily living (IADL) were used to evaluate the functional limitation [41–43]. The ADL limitation referred to the difficulty in bathing, dressing, feeding oneself, using the toilet, getting in or out of bed, and controlling urination and defecation. The IADL limitation referred to the difficulty in doing household chores, cooking, shopping, making telephone calls, taking medications, and managing finances. For both ADL and IADL, answers were categorized as: ‘can do it by myself’, ‘have some difficulties’, ‘need help’ and ‘cannot do it’. Binary variables were constructed, so ADL/ IADL disability was defined as having difficulty in one or more ADL/IADL items. For the multivariable regression analysis, we used binary variables for no difficulty, and one or more difficulties.

The second outcome measure, work productivity loss, had four variables: (1) limited work due to health problems, which was a binary variable and was derived from the question ‘Are you unable to do some kind of work or cannot do that work for a long time because of a disability or health reasons?’; (2) early retirement, which was a binary variable and was derived from the question ‘Have you completed retirement procedures including early retirement and/or internal retirement?’; (3) number of days missed due to sick leave at agricultural work and (4) sick leave of employee’s primary non-agricultural work (including self-employed work), which was calculated based on the questions ‘How many days of work did you miss last year due to a health problem’ [32].

Covariates included age, gender, marital status (married and partnered, unmarried and others), level of education (illiterate, primary school, secondary school, college and above), place of residence (rural, urban), household economic status quartiles (yearly per capita household consumption expenditure), social health insurance (yes, no) and geographical region (east, central, west). The Central and West referred to more deprived regions than the East with a high level of economic development in China.

### Statistical analysis

We summarised the characteristics of all samples and the prevalence of multimorbidity. Means, proportion and 95% confidence intervals (CIs) for functional limitations and work productivity loss were calculated separately for individuals with different numbers of diseases (zero, one, two and above). Multivariable logistic regression models were performed to examine the impact of multimorbidity on functional limitations, limited work due to health problems, and early retirement. Negative binomial regression models were used to assess the association of multimorbidity with the number of days of sick leave at agricultural work and employed non-agricultural work. Moreover, we investigated the urban-rural differences in the relationship between multimorbidity and these outcomes. For the logistic regression analyses, the adjusted odds ratio (OR) and 95% confidence intervals (CIs) were reported. For the negative binomial regression analysis, we reported the incidence rate ratio (IRR) and 95% CIs.

We also performed sensitivity analyses to examine the association of the number of chronic diseases with functional health and work productivity loss using zero disease as a reference group (Appendix table S1, table S2 and table S3). Multivariable regressions also further adjusted for the Body Mass Index (BMI) [≥18.5 to <25.0, <18.5, ≥25.0 to <30.0, ≥30.0], depressive symptoms (no, yes), physical activity (low level, moderate level, high level). All descriptive analyses and regression results were weighted to account for the complex, multi-stage design, and non-response in the CHARLS data. All statistical analyses were conducted using Stata software 15.0 (Stata Corp., College Station, Texas). P values less than 0.05 were considered statistically significant.

## Results

We analysed data from 11,167 respondents from CHARLS 2015. Table 1 shows sample characteristics and the prevalence of single disease and multimorbidity across demographic groups. The mean age of respondents was 60.6 years. 43.2% of the respondents were illiterate, 63.3% resided in rural areas and 85.1% had social health insurance. A high prevalence of 68.8% had multimorbidity, which ranged from 62.0% in those aged 45–54 years, and 75.3% for those aged ≥ 75 years. The prevalence of multimorbidity was higher among individuals insured by social health insurance (77.4%) compared to people without health insurance (67.2%).

**Table 1.** Characteristics of participants by number of chronic diseases

Characteristic	Total sample		Zero disease			One disease			Multimorbidity		
	N	%	%	95% CI		%	95% CI		%	95% CI	
Total	11,167	100.0	7.8	7.2	8.4	23.5	22.4	24.5	68.8	67.6	69.9
Age (year)											
45–54	3,358	30.1	8.8	7.8	10.0	29.2	27.0	31.6	62.0	59.6	64.3
55–64	4,134	37.0	8.7	7.7	9.8	22.2	20.7	23.8	69.1	67.3	70.8
65–74	2,788	25.0	5.9	5.1	6.9	19.7	18.1	21.5	74.4	72.5	76.2
75 and above	8,96	8.0	5.5	3.9	7.6	19.2	16.3	22.4	75.3	71.8	78.6
Gender											
Male	5,285	47.3	7.4	6.6	8.2	24.3	22.7	26.0	68.3	66.6	70.0
Female	5,891	52.7	8.1	7.3	9.0	22.7	21.4	24.0	69.2	67.7	70.6
Marital status											
Married and partnered	9,753	87.3	8.0	7.4	8.6	23.7	22.6	24.8	68.3	67.1	69.5
Unmarried and other	1,423	12.7	6.4	5.1	8.1	22.0	19.6	24.5	71.6	68.8	74.2
Education status											
Illiterate	4,828	43.2	7.3	6.5	8.2	23.0	21.6	24.4	69.8	68.3	71.2
Primary school	2,911	26.1	5.8	4.9	6.9	20.0	18.3	21.8	74.2	72.2	76.0
Secondary school	2,293	20.5	10.1	8.7	11.6	26.7	24.2	29.3	63.3	60.5	65.9
College & above	1,144	10.2	9.6	7.9	11.6	26.8	23.1	30.9	63.6	59.5	67.6
Residence place											
Urban	4,103	36.7	6.8	5.9	7.8	22.6	20.8	24.5	70.6	68.5	72.6
Rural	7,073	63.3	8.5	7.9	9.2	24.1	23.1	25.2	67.3	66.1	68.5
Region											
East	4,163	37.3	8.8	7.8	9.9	25.1	23.2	27.1	66.1	63.9	68.1
Central	4,278	38.3	7.4	6.6	8.4	21.7	20.3	23.2	70.8	69.3	72.4
West	2,735	24.5	6.5	5.6	7.6	23.4	21.6	25.2	70.1	68.2	72.0
PCE, quartile											
Q1, the lowest	2,803	25.1	9.0	7.8	10.3	22.5	20.8	24.3	68.5	66.5	70.5
Q2	2,785	24.9	8.0	6.9	9.3	26.0	23.9	28.3	66.0	63.6	68.2
Q3	2,794	25.0	7.1	6.2	8.2	22.2	20.4	24.2	70.7	68.6	72.7
Q4 (the highest)	2,794	25.0	7.2	6.1	8.5	23.1	21.0	25.4	69.7	67.2	72.0
Social health insurance											
No	1,664	14.9	4.5	3.4	5.8	18.2	15.8	20.8	77.4	74.6	79.9
Yes	9,512	85.1	8.4	7.8	9.0	24.4	23.3	25.5	67.2	66.0	68.4

The values are unweighted counts and weighted percentages unless otherwise indicated.

### Functional disability and productivity loss by the number of conditions and place of residence

Table 2 displayed the average proportion of subjects with ADL, IADL, limited work due to health problems, and early retirement (19.2%, 21.9%, 14.1% and 17.3%, respectively). The mean number of days of sick leave for agricultural work and employed non-agricultural works were 17.0 days and 2.5 days, respectively. The proportions of or mean of these outcomes increased as the number of conditions increased. Rural residents with multimorbidity reported higher proportions of physical functions and days of sick leave than urban residents.

**Table 2.** Functional limitations and productivity loss by number of chronic diseases and residence

Variables	Total			Rural			Urban		
	Mean	95% CI		Mean	95% CI		Mean	95% CI	
Limitation of ADL, %									
Overall	19.2	18.3	20.2	22.6	21.5	23.6	14.9	13.3	16.5
Zero	7.5	5.7	9.3	8.3	6.1	10.6	6.1	3.1	9.1
One	12.6	11.2	14.0	15.8	13.9	17.7	8.1	6.1	10.0
Multimorbidity	22.8	21.6	24.1	26.8	25.5	28.2	17.9	15.8	20.1
Limitation of IADL, %									
Overall	21.9	20.9	22.8	26.6	25.5	27.7	15.8	14.2	17.4
Zero	14.3	11.9	16.7	18.5	15.3	21.7	7.6	4.4	10.8
One	16.5	15.0	18.1	20.7	18.5	22.8	10.9	8.8	12.9
Multimorbidity	24.6	23.3	25.8	29.8	28.4	31.2	18.2	16.0	20.3
Limited work due to health problems, %									
Overall	14.1	13.3	14.8	17.4	16.4	18.3	9.8	8.8	10.8
Zero	6.0	4.1	7.9	7.7	4.9	10.5	3.4	1.4	5.3
One	9.7	8.5	10.9	12.3	10.6	14.0	6.1	4.5	7.6
Multimorbidity	16.5	15.5	17.4	20.4	19.2	21.7	11.6	10.3	12.9
Early retirement, %									
Overall	17.3	14.3	20.3	18.3	13.7	23.0	17.1	13.7	20.5
Zero	14.2	6.2	22.2	6.2	−5.7	18.1	15.2	6.3	24.1
One	17.5	12.2	22.8	16.5	6.8	26.1	17.7	11.7	23.6
Multimorbidity	17.4	13.7	21.2	19.6	14.1	25.2	17.1	12.7	21.4
Sick leave at agricultural work, days									
Overall	17.0	15.7	18.2	18.4	16.9	19.9	11.6	9.5	13.7
Zero	10.2	6.1	14.2	11.2	6.4	16.0	5.1	1.1	9.1
One	11.4	9.6	13.2	12.0	9.9	14.1	9.1	5.8	12.3
Multimorbidity	20.2	18.5	21.8	22.0	20.0	24.0	13.3	10.4	16.1
Sick leave at employed non-agricultural work, days									
Overall	2.5	1.8	3.3	2.6	1.5	3.8	2.4	1.5	3.4
Zero	1.7	−0.5	3.9	1.5	−0.8	3.8	1.9	−1.5	5.3
One	2.0	0.6	3.5	2.5	−0.3	5.4	1.6	0.6	2.5
Multimorbidity	2.8	1.9	3.8	2.9	1.6	4.2	2.8	1.6	4.1

The employed non-agricultural work includes self-employed non-agricultural work.

**Figure 1.**
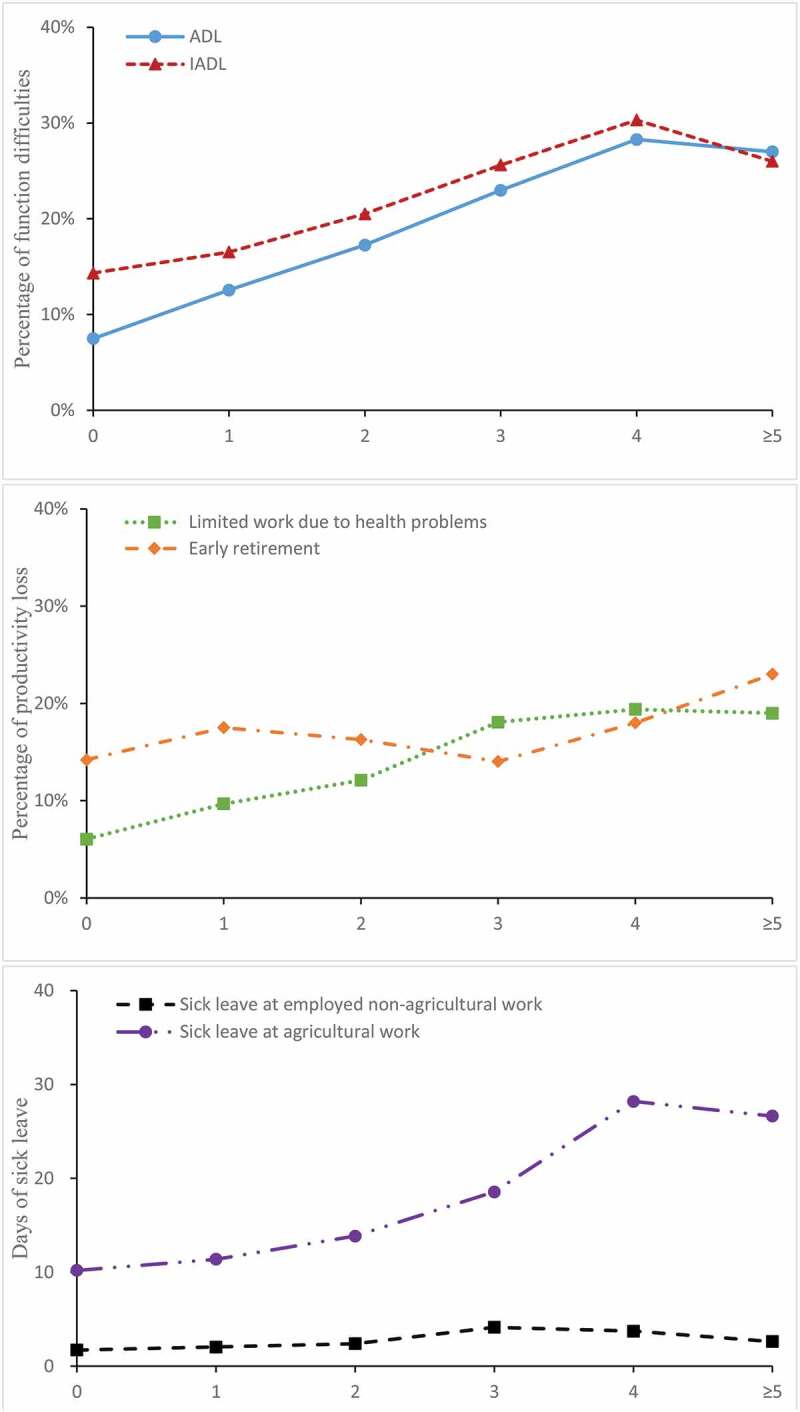
The percentage of functional difficulties and productivity loss among Chinese adults in 2015, by number of chronic diseases

The proportion of subjects with ADL limitation and IADL limitation rose as the number of NCDs increased from 0 to 4, but decreased slightly as the number of NCDs was 5 and above, which indicated a positive association of an increased number of co-morbidity and deteriorating physical and instrumental functions. Similarly, the proportion of limited work due to health problems, days of sick leave at agricultural work and employed non-agricultural work increased as the number of NCDs increased and then declined. Number of days of sick leave were greater in individuals at agricultural work than those having non-agricultural work. There was no linear association between multimorbidity and early retirement. (Figure 1)

### Association of multimorbidity with function difficulties and productivity loss

Compared with subjects with a single disease, patients with multimorbidity had a higher likelihood of ADL limitation (OR = 1.924, 95% CI = 1.656, 2.236) and IADL limitation (OR = 1.522, 95% CI = 1.326, 1.748). Meanwhile, participants aged 55 years and over were found to have greater odds of physical function limitations, compared to those aged below 55 years. Participants who were females, had lower education, from rural residences and deprived regions in China had greater odds of ADL and IADL. (Table 3)

**Table 3.** Association of functional limitations with multimorbidity and socio-demographic factors

Variables	Limitation of ADL				Limitation of IADL			
	OR	P value	95% CI		OR	P value	95% CI	
No. of chronic disease (single)								
Multimorbidity	1.924	<0.001	1.656	2.236	1.522	<0.001	1.326	1.748
Age (45–54)								
55–64	1.633	<0.001	1.306	2.042	1.775	<0.001	1.430	2.202
65–74	2.314	<0.001	1.917	2.793	2.563	<0.001	2.170	3.027
75 and above	3.234	<0.001	2.402	4.354	4.436	<0.001	3.362	5.854
Gender (male)								
Female	1.445	<0.001	1.262	1.654	1.908	<0.001	1.685	2.160
Marital status (married)								
Unmarried and other	1.140	0.157	0.951	1.367	1.056	0.505	0.899	1.241
Level of education (illiterate)								
Primary school	0.764	0.003	0.638	0.914	0.519	<0.001	0.437	0.615
Secondary school	0.674	<0.001	0.569	0.799	0.451	<0.001	0.373	0.545
College & above	0.465	<0.001	0.348	0.620	0.242	<0.001	0.177	0.332
Residence place (urban)								
Rural	1.496	<0.001	1.268	1.765	1.520	<0.001	1.291	1.788
Region (east)								
Central	1.700	<0.001	1.430	2.020	1.539	<0.001	1.306	1.814
West	1.334	0.005	1.093	1.628	1.560	<0.001	1.277	1.906
PCE, quartile (Q1, the lowest)								
Q2	1.025	0.796	0.849	1.237	0.895	0.204	0.755	1.062
Q3	0.910	0.338	0.750	1.104	0.798	0.015	0.666	0.957
Q4 (the highest)	0.851	0.064	0.717	1.010	0.768	0.002	0.651	0.906
Social health insurance (no)								
Yes	1.238	0.026	1.025	1.494	1.166	0.086	0.978	1.390

Logistic regression models were used to examine the association of functional limitations with chronic conditions. The odds ratios estimated by adjusting for study variables, including age, gender, marital status, level of education, residence place, region, household economic level and health insurance status. ADL, activities of daily living; IADL, instrumental activities of daily living; OR, odds ratio; CI, confidence interval; PCE, per capita household consumption expenditure.

Table 4 showed the association between the number of chronic conditions and work productivity loss. Compared with individuals with a single disease, people with multimorbidity were more likely to be unable to do certain types of work or unable to do that type of work for a long time (limited work due to health problems: OR = 1.868, 95% CI = 1.601, 2.178). Subjects living in rural areas were more likely to be unable to do certain types of work or unable to do that type of work for a long time due to health problems (limited work due to health problems: OR = 1.715, 95% CI = 1.457, 2.018), compared to those in urban areas. Additionally, the education level and health insurance were risk factors of limited work due to health problems. Compared to those with a single disease, individuals with multimorbidity did not have significantly higher odds of having early retirement (P > 0.05).

**Table 4.** Association of work productivity loss with multimorbidity and socio-demographic factors

Variables	Limited work due to health problems (n = 10,261)				Early retirement (n = 1,289)			
	OR	P value	95% CI		OR	P value	95% CI	
No. of chronic disease (single)								
Multimorbidity	1.868	<0.001	1.601	2.178	1.090	0.681	0.721	1.648
Age (45–54)								
55–64	1.062	0.462	0.905	1.245	0.551	0.052	0.301	1.006
65–74	1.012	0.898	0.843	1.214	0.636	0.277	0.281	1.439
75 and above	0.809	0.162	0.601	1.089	0.264	0.001	0.118	0.592
Gender (male)								
Female	1.082	0.204	0.958	1.223	0.858	0.576	0.500	1.471
Marital status (married)								
Unmarried and other	1.167	0.130	0.956	1.424	1.125	0.710	0.604	2.094
Level of education (illiterate)								
Primary school	1.024	0.768	0.874	1.200	1.187	0.585	0.641	2.197
Secondary school	0.813	0.019	0.683	0.966	2.071	0.035	1.054	4.068
College & above	0.585	<0.001	0.443	0.774	1.927	0.024	1.091	3.403
Residence place (urban)								
Rural	1.715	<0.001	1.457	2.018	1.110	0.643	0.712	1.731
Region (east)								
Central	1.287	0.003	1.091	1.519	1.239	0.262	0.851	1.805
West	1.134	0.194	0.938	1.372	1.926	0.022	1.100	3.372
PCE, quartile (Q1, the lowest)								
Q2	1.012	0.892	0.856	1.195	1.121	0.737	0.574	2.188
Q3	0.838	0.056	0.698	1.005	0.631	0.175	0.324	1.229
Q4 (the highest)	0.683	<0.001	0.564	0.827	1.169	0.631	0.618	2.210
Social health insurance (no)								
Yes	1.296	0.010	1.065	1.576	1.134	0.677	0.626	2.057

Logistic regression models were used to assess the association of limited work due to health problems and early retirement with chronic conditions. The odds ratios estimated by adjusting for study variables, including age, gender, marital status, level of education, residence place, region, household economic level and health insurance status. OR, odds ratio; CI, confidence interval; PCE, per capita household consumption expenditure.

As shown in Table 5, after controlling covariates, compared to those with a single condition, individuals with multimorbidity had more days of sick leave at agricultural work (IRR = 1.676, 95% CI = 1.390, 2.020), and non-agricultural work (IRR = 2.418, 95% CI = 1.245, 4.696).

**Table 5.** Association of days of sick leave with multimorbidity and socio-demographic factors

Variables	Sick leave at agricultural work (n = 4,965)				Sick leave at employed non-agricultural work (n = 1,006)			
	IRR	P value	95% CI		IRR	P value	95% CI	
No. of chronic disease (single)								
Multimorbidity	1.676	<0.001	1.390	2.020	2.418	0.009	1.245	4.696
Age (45–54)								
55–64	1.111	0.277	0.919	1.343	0.671	0.201	0.364	1.238
65–74	1.331	0.010	1.072	1.654	-	-	-	-
−75 and above	1.259	0.231	0.863	1.835	-	-	-	-
Gender (male)								
Female	1.272	0.004	1.080	1.498	0.740	0.297	0.419	1.306
Marital status (married)								
Unmarried and other	1.041	0.782	0.784	1.382	2.442	0.181	0.658	9.056
Level of education (illiterate)								
Primary school	0.998	0.985	0.809	1.232	2.185	0.073	0.930	5.131
Secondary school	0.702	0.002	0.562	0.876	2.366	0.007	1.262	4.437
College & above	0.830	0.389	0.542	1.270	1.422	0.387	0.640	3.160
Residence place (urban)								
Rural	1.610	<0.001	1.301	1.993	1.011	0.969	0.589	1.736
Region (east)								
Central	1.135	0.237	0.920	1.399	0.923	0.801	0.496	1.719
West	1.326	0.008	1.076	1.633	0.746	0.403	0.376	1.483
PCE, quartile (Q1, the lowest)								
Q2	0.895	0.276	0.734	1.093	1.073	0.901	0.350	3.291
Q3	1.179	0.093	0.973	1.429	0.677	0.433	0.254	1.801
Q4 (the highest)	1.125	0.267	0.913	1.385	1.444	0.390	0.623	3.345
Social health insurance (no)								
Yes	1.146	0.337	0.867	1.515	1.778	0.115	0.869	3.640

Negative binomial regression models were used to assess the association between days of sick leave at agricultural work and at employed non-agricultural work with chronic conditions. The incidence rate ratio estimated by adjusting for study variables, including age, gender, marital status, level of education, residence place, region, household economic level and health insurance status. IRR, incidence rate ratio; CI, confidence interval; PCE, per capita household consumption expenditure.

**Figure 2.**
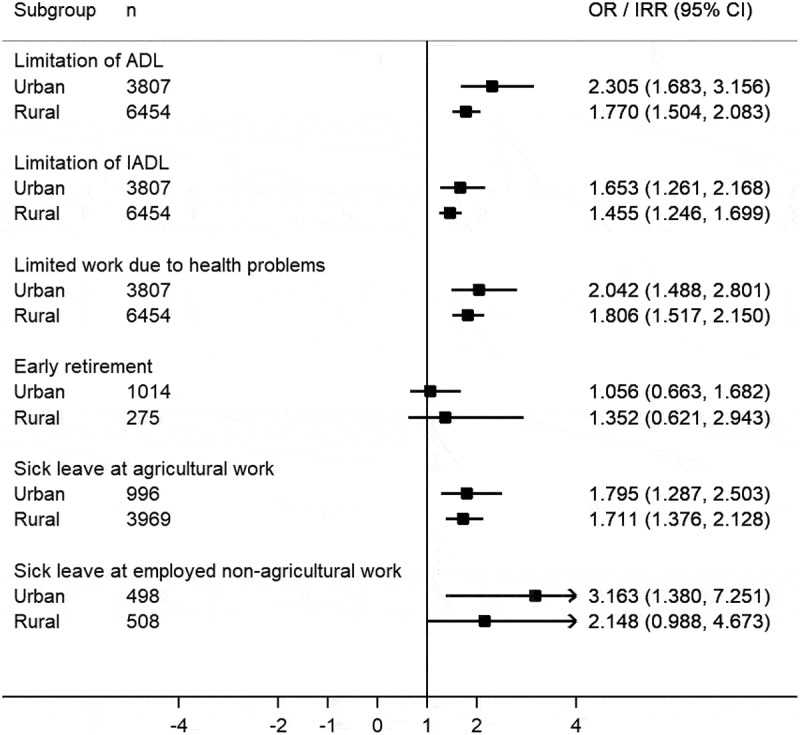
Association of multimorbidity with functional limitations and productivity loss by residence place

Overall, the urban-rural differences in the association of multimorbidity with functional health and work productivity loss were comparable to the result of the whole regression analyses. Compared to the rural group, those in the urban group had a lower impact of multimorbidity on ADL limitation and IADL limitation. Moreover, except for the outcome on having early retirement, there were similar urban-rural differences in the relationships between multimorbidity and limited work due to health problems, as well as number of days of sick leave. (Figure 2)

### Sensitivity analysis

Compared with individuals in the zero-disease group, subjects with multimorbidity were at a higher likelihood of ADL limitation (OR = 3.283, 95% CI = 2.395, 4.498), IADL limitation (O = 1.714, 95% CI = 1.381, 2.126), and limited work due to health problems (OR = 2.955, 95% CI = 2.097, 4.165). Compared to those with a single disease, individuals with multiple chronic diseases did not have significantly higher odds of having early retirement. (Table S1 and Table S2) Individuals with multimorbidity compared to those people with zero disease are expected to have a 1.891 times more days of sick leave at agricultural work. (Table S3). After further adjusting for BMI, mental condition and physical activity in multivariable regression models, Table S4, Table S5 and Table S6 also showed consistent associations of multimorbidity with functional limitations and work productivity loss reported before.

## Discussion

### Summary of findings

Our findings demonstrate that there is a high prevalence of physical multimorbidity among middle-aged and elderly Chinese adults (68.8%), with the oldest age-groups having the greatest prevalence (75.3%). Multimorbidity has become a major health issue for the majority of Chinese adults and its prevalence increases with age.

Our findings also show that physical multimorbidity is associated with increased functional difficulties in daily life and instrumental daily life. This finding is consistent with our previous work in China [14,22], and research in USA [44,45], Japan [46], and European countries [47], where a higher number of chronic diseases were present for ADL and IADL difficulty. The decrease in physical functioning inevitably leads to an increased utilization of health services. This is then also associated with increasing health expenditure as well as an an increased burden on individuals, their families and the wider society [33,48,49]. Quah and colleagues examined multimorbid older patients in Singapore and they highlighted the need for an enhanced model of primary care to address the quality of life and mental health demands [50]. A strong primary care system needs to address multimorbidity. Universal health coverage for patients with multimorbidity has been proven to be cost-effective [51,52]. The improvement of patients’ self-efficacy and self-management under the guidance of primary care physicians, and the assessment and change of lifestyle behaviors is also proposed as an effective intervention in curbing the development of chronic conditions, while also maintaining physical functioning and emotional health [53–55].

This study has also identified an association between physical multimorbidity and a higher number of sick-leave days at employed non-agricultural work as well as at agricultural work, with a greater effect on non-agricultural work. These findings are consistent with the small number of existing studies that have examined the association of multiple chronic diseases with a reduction in productivity in China [14,22], USA [44,45] and European countries [47]. This could be due to social welfare for formally employed workers, such as social and endowment insurance, employee medical insurance or sick leave assistance, which are not available to those people engaged in agricultural production.

Importantly, this study suggests that there is less impact of multimorbidity on functional limitations and work productivity among rural participants, compared to those living in urban areas. This is because people in rural areas are relatively poorer than urban residents and may have no choice but to keep working due to limited economic means. The urban-rural differences were probably because of the greater proportion of functional difficulties and productivity loss among the single disease group in rural areas than urban areas (e.g. for ADL limitation, 15.8% vs 8.1%; for limited work due to health problem, 12.3% vs 6.1%). It indicates that the single disease could have a greater impact on functional health, limited work and sick leave to rural residents compared to urban citizens. These findings have implications for practice and policy, suggesting that prevention and self-management programs in rural areas should be targeted to decrease the incidence of any chronic disease, to delay high economic burden. Our results did not show an association between multimorbidity and early retirement which may be due to the lower age-group (45 to 60 years) of subjects that are not at retirement age yet.

### Strengths and limitations

To our knowledge, this is the first study to examine the rural-urban differences in the relationship between physical multimorbidity and functional difficulties and work productivity loss in China. However, our study has some important limitations. First, the use of self-reported measures of chronic conditions may underestimate their prevalence, particularly for older persons and those from lower socio-economic and educational backgrounds who may be more likely to under-report. Second, previous systematic reviews found that in individuals without diabetes mellitus, HbA1c values are higher in Asians and Latinos when compared to persons with a Caucasian background [56]. Using the diagnostic criterion by the American Diabetes Association (HbA1c concentration of ≥6.5%) for diabetes could potentially cause bias, but this bias is also minimised because the current study used three different criteria for the diabetes diagnosis. Third, we examined the effect of multimorbidity on productivity loss by just counting the number of chronic diseases without accounting for the different clusters and severity of chronic diseases.Future research should also consider mental health problems because effects of the combination of physical and mental health conditions on work ability tend to be greater than physical health problems alone, Fourth, the presence of unobserved determinants (i.e. residual confounding) biases the estimation in this study, and the directional or causal relationship between multimorbidity and outcomes (i.e. functional impairment) could not established with the cross-sectional design [57]. Finally, this study only included middle-aged and older populations in China, which may limit the generalizability of the study. The prevalence of multimorbidity and its impacts on cost in terms of productivity loss among younger populations, as well as prospective study designs, should be considered for future studies.

### Policy implications

Our findings provide new evidence on the burden of functional disability and the growing productivity loss due to multimorbidity in China. Multimorbidity is costly, not only to individuals and households, but also for the wider society, which is a major unaddressed challenge to health systems in China and other LMICs. Concerted efforts are needed in China with the largest ageing population in the world. Healthcare systems need to shift from single-disease models to new financing and service delivery models to more effectively manage multimorbidity [58]. A strong primary healthcare system, behaviour monitoring, and behavioural change counselling led by more generalist physicians (less over-specialization) working with multi-disciplinary teams, is essential for cost-effective management of multimorbidity [24,55,59].

Preventive strategies for multimorbidity can have substantial returns on health benefits as well as work productivity gains. Transforming the workplace environment might be a great preventive strategy to incorporate. By utilising strategies that maintain and improve the ability to work and secure sustainable employability for employees with multiple chronic conditions, such as providing on-site counselling services and worksite exercise space, businesses can reduce the effects that employees’ health conditions have on their productivity [60–62].

A few studies from other countries have suggested that comorbid mental-physical health problems have reportedly led to a significant increase in disability and productivity loss [13,20]. Such interactions between mental conditions and physical multimorbidity are indirectly consistent with evidence suggesting that comorbid mental disorders can complicate the management of and exacerbate the course of chronic physical conditions [63]. Future research in China is needed to examine the joint effects of mental-physical health conditions on work impairment.

Also, public health interventions should be better designed to consider physical-mental multimorbidity and be targeted to enable individuals with multimorbidity to improve functional health, job well-being and reduce absence from work. Such interventions should also address rural-urban disparities by accounting for elevated risks associated with functional disabilities and reduced labour force participation among rural residents. Future research related to mental-physical multimorbidity is also needed to enable the development of interventions that can be applied to the wider community.

## Conclusion

Chronic disease multimorbidity is common among middle-aged and elderly Chinese adults. Multimorbidity poses health challenges and adversely affects productivity, which has negative economic consequences for individuals, the health system, and the wider society in China. Strategies for better management and control of multimorbidity could potentially improve the functional health of those affected as well as lead to productivity gains in China.

## Supplementary Material

Supplemental Material

## Paper context

Previous research has evaluated the direct economic burden of multimorbidity on health care utilization and cost. This nationally representative population-based study provides new evidence on the greater burden of functional disability and growing productivity loss due to multimorbidity in China, which is higher in rural residents than urban residents. Healthcare systems need to shift from single-disease models to new financing and service delivery models to more effectively manage multimorbidity and also address rural-urban disparities.
