# Supplementary material for: Urban-rural differences in the impacts of multiple chronic disease on functional limitations and work productivity among Chinese adults
Source: Glob Health Action. 2021 Sep 17;14(1):1975921. doi: 10.1080/16549716.2021.1975921 (PMC8451617; doi:10.1080/16549716.2021.1975921)
Supplement: Supplemental Material [file ZGHA_A_1975921_SM3370.docx]

**Supplementary Appendix:**

**Table S1** Association of functional limitations with chronic conditions and socio-demographic factors

| Variables | Limitation of ADL | | | | |  | Limitation of IADL | | | | |
| --- | --- | --- | --- | --- | --- | --- | --- | --- | --- | --- | --- |
|  | OR | | P value | 95% CI | |  | OR | | P value | 95% CI | |
| No. of chronic disease (zero) |  |  | |  |  |  |  |  | |  |  |
| One | 1.841 | <0.001 | | 1.331 | 2.546 |  | 1.224 | 0.092 | | 0.968 | 1.547 |
| Two and above | 3.543 | <0.001 | | 2.590 | 4.846 |  | 1.863 | <0.001 | | 1.505 | 2.306 |
| Age (45-54) |  |  | |  |  |  |  |  | |  |  |
| 55-64 | 1.621 | <0.001 | | 1.305 | 2.013 |  | 1.773 | <0.001 | | 1.443 | 2.178 |
| 65-74 | 2.296 | <0.001 | | 1.910 | 2.760 |  | 2.606 | <0.001 | | 2.218 | 3.062 |
| 75 and above | 3.180 | <0.001 | | 2.381 | 4.247 |  | 4.370 | <0.001 | | 3.347 | 5.707 |
| Gender (male) |  |  | |  |  |  |  |  | |  |  |
| Female | 1.451 | <0.001 | | 1.272 | 1.655 |  | 1.853 | <0.001 | | 1.646 | 2.087 |
| Marital status (married) |  |  | |  |  |  |  |  | |  |  |
| Unmarried and other | 1.159 | 0.100 | | 0.972 | 1.382 |  | 1.042 | 0.613 | | 0.888 | 1.222 |
| Level of education (illiterate) |  |  | |  |  |  |  |  | |  |  |
| Primary school | 0.777 | 0.005 | | 0.651 | 0.928 |  | 0.512 | <0.001 | | 0.435 | 0.604 |
| Secondary school | 0.679 | <0.001 | | 0.576 | 0.801 |  | 0.441 | <0.001 | | 0.368 | 0.529 |
| College & above | 0.481 | <0.001 | | 0.362 | 0.641 |  | 0.245 | <0.001 | | 0.180 | 0.334 |
| Residence place (urban) |  |  | |  |  |  |  |  | |  |  |
| Rural | 1.490 | <0.001 | | 1.269 | 1.751 |  | 1.546 | <0.001 | | 1.320 | 1.811 |
| Region (east) |  |  | |  |  |  |  |  | |  |  |
| Central | 1.687 | <0.001 | | 1.428 | 1.993 |  | 1.515 | <0.001 | | 1.290 | 1.779 |
| West | 1.325 | 0.005 | | 1.091 | 1.609 |  | 1.572 | <0.001 | | 1.292 | 1.913 |
| PCE, quartile (Q1, the lowest) |  |  | |  |  |  |  |  | |  |  |
| Q2 | 1.010 | 0.914 | | 0.844 | 1.209 |  | 0.889 | 0.157 | | 0.756 | 1.047 |
| Q3 | 0.892 | 0.230 | | 0.740 | 1.075 |  | 0.818 | 0.024 | | 0.687 | 0.974 |
| Q4 (the highest) | 0.843 | 0.044 | | 0.714 | 0.995 |  | 0.762 | 0.001 | | 0.651 | 0.892 |
| Social health insurance (no) |  |  | |  |  |  |  |  | |  |  |
| Yes | 1.239 | 0.025 | | 1.028 | 1.493 |  | 1.153 | 0.103 | | 0.972 | 1.367 |

**Notes:** Logistic regression models were used to examine the association of functional limitations with chronic conditions. The odds ratios estimated by adjusting for study variables, including age, gender, marital status, level of education, residence place, region, household economic level and health insurance status. ADL, activities of daily living; IADL, instrumental activities of daily living; OR, odds ratio; CI, confidence interval; PCE, Per capita household consumption expenditure.

**Table S2** Association of work productivity loss with chronic conditions and socio-demographic factors

| Variables | Limited work due to health problems (n=11,176) | | | | |  | Early retirement  (n=1,376) | | | |
| --- | --- | --- | --- | --- | --- | --- | --- | --- | --- | --- |
|  | OR | | P value | 95% CI | |  | OR | P value | 95% CI | |
| No. of chronic disease (zero) |  |  | |  |  |  |  |  |  |  |
| One | 1.706 | 0.003 | | 1.204 | 2.418 |  | 1.315 | 0.485 | 0.608 | 2.847 |
| Two and above | 3.186 | <0.001 | | 2.275 | 4.462 |  | 1.423 | 0.322 | 0.707 | 2.865 |
| Age (45-54) |  |  | |  |  |  |  |  |  |  |
| 55-64 | 1.068 | 0.411 | | 0.912 | 1.251 |  | 0.567 | 0.059 | 0.315 | 1.023 |
| 65-74 | 1.012 | 0.899 | | 0.847 | 1.209 |  | 0.653 | 0.295 | 0.293 | 1.453 |
| 75 and above | 0.839 | 0.247 | | 0.623 | 1.130 |  | 0.266 | 0.001 | 0.122 | 0.582 |
| Gender (male) |  |  | |  |  |  |  |  |  |  |
| Female | 1.075 | 0.250 | | 0.950 | 1.215 |  | 0.805 | 0.426 | 0.472 | 1.374 |
| Marital status (married) |  |  | |  |  |  |  |  |  |  |
| Unmarried and other | 1.197 | 0.075 | | 0.982 | 1.458 |  | 1.141 | 0.673 | 0.617 | 2.111 |
| Level of education (illiterate) |  |  | |  |  |  |  |  |  |  |
| Primary school | 1.006 | 0.939 | | 0.859 | 1.178 |  | 1.178 | 0.582 | 0.656 | 2.115 |
| Secondary school | 0.804 | 0.012 | | 0.679 | 0.953 |  | 2.056 | 0.032 | 1.064 | 3.972 |
| College & above | 0.595 | <0.001 | | 0.454 | 0.781 |  | 1.871 | 0.026 | 1.078 | 3.248 |
| Residence place (urban) |  |  | |  |  |  |  |  |  |  |
| Rural | 1.731 | <0.001 | | 1.472 | 2.034 |  | 1.030 | 0.895 | 0.659 | 1.611 |
| Region (east) |  |  | |  |  |  |  |  |  |  |
| Central | 1.260 | 0.006 | | 1.070 | 1.483 |  | 1.277 | 0.194 | 0.883 | 1.849 |
| West | 1.116 | 0.255 | | 0.924 | 1.349 |  | 2.182 | 0.008 | 1.222 | 3.896 |
| PCE, quartile (Q1, the lowest) |  |  | |  |  |  |  |  |  |  |
| Q2 | 1.004 | 0.961 | | 0.848 | 1.188 |  | 1.073 | 0.832 | 0.561 | 2.051 |
| Q3 | 0.843 | 0.061 | | 0.706 | 1.008 |  | 0.626 | 0.152 | 0.329 | 1.189 |
| Q4 (the highest) | 0.693 | <0.001 | | 0.574 | 0.837 |  | 1.146 | 0.666 | 0.616 | 2.130 |
| Social health insurance (no) |  |  | |  |  |  |  |  |  |  |
| Yes | 1.293 | 0.009 | | 1.068 | 1.566 |  | 1.163 | 0.619 | 0.641 | 2.110 |

**Notes:** Logistic regression models were used to assess the association of limited work due to health problems and early retirement with chronic conditions. The odds ratios estimated by adjusting for study variables, including age, gender, marital status, level of education, residence place, region, household economic level and health insurance status. OR, odds ratio; CI, confidence interval; PCE, Per capita household consumption expenditure.

**Table S3** Association of days of sick leave with chronic conditions and socio-demographic factors

| Variables | Sick leave at agricultural work  (n=5,488) | | | |  | Sick leave at employed non-agricultural work (n=1,128) | | | |
| --- | --- | --- | --- | --- | --- | --- | --- | --- | --- |
|  | IRR | P value | 95% CI | |  | IRR | P value | 95% CI | |
| No. of chronic disease (zero) |  |  |  |  |  |  |  |  |  |
| One | 1.119 | 0.612 | 0.725 | 1.726 |  | 1.318 | 0.631 | 0.426 | 4.079 |
| Two and above | 1.891 | 0.003 | 1.253 | 2.855 |  | 3.172 | 0.052 | 0.988 | 10.184 |
| Age (45-54) |  |  |  |  |  |  |  |  |  |
| 55-64 | 1.194 | 0.096 | 0.969 | 1.472 |  | 0.574 | 0.074 | 0.312 | 1.055 |
| 65-74 | 1.373 | 0.004 | 1.108 | 1.700 |  | - | - | - | - |
| -75 and above | 1.526 | 0.031 | 1.039 | 2.242 |  | - | - | - | - |
| Gender (male) |  |  |  |  |  |  |  |  |  |
| Female | 1.231 | 0.012 | 1.046 | 1.449 |  | 0.850 | 0.565 | 0.488 | 1.481 |
| Marital status (married) |  |  |  |  |  |  |  |  |  |
| Unmarried and other | 1.001 | 0.995 | 0.765 | 1.310 |  | 2.031 | 0.246 | 0.613 | 6.736 |
| Level of education (illiterate) |  |  |  |  |  |  |  |  |  |
| Primary school | 0.940 | 0.562 | 0.762 | 1.160 |  | 2.120 | 0.091 | 0.887 | 5.063 |
| Secondary school | 0.759 | 0.032 | 0.591 | 0.976 |  | 2.369 | 0.009 | 1.240 | 4.525 |
| College & above | 0.773 | 0.220 | 0.511 | 1.168 |  | 1.806 | 0.168 | 0.778 | 4.189 |
| Residence place (urban) |  |  |  |  |  |  |  |  |  |
| Rural | 1.595 | <0.001 | 1.301 | 1.955 |  | 0.960 | 0.875 | 0.577 | 1.596 |
| Region (east) |  |  |  |  |  |  |  |  |  |
| Central | 1.022 | 0.839 | 0.831 | 1.255 |  | 0.849 | 0.587 | 0.471 | 1.532 |
| West | 1.291 | 0.037 | 1.016 | 1.640 |  | 0.766 | 0.403 | 0.410 | 1.432 |
| PCE, quartile (Q1, the lowest) |  |  |  |  |  |  |  |  |  |
| Q2 | 0.968 | 0.784 | 0.766 | 1.223 |  | 1.055 | 0.918 | 0.377 | 2.955 |
| Q3 | 1.187 | 0.079 | 0.980 | 1.438 |  | 0.678 | 0.409 | 0.269 | 1.710 |
| Q4 (the highest) | 1.108 | 0.337 | 0.898 | 1.368 |  | 1.865 | 0.122 | 0.845 | 4.113 |
| Social health insurance (no) |  |  |  |  |  |  |  |  |  |
| Yes | 1.120 | 0.411 | 0.854 | 1.469 |  | 1.749 | 0.144 | 0.826 | 3.703 |

**Notes:** Negative binomial regression models were used to assess the association between days of sick leave at agricultural work and at employed non-agricultural work with chronic conditions. The incidence rate ratio estimated by adjusting for study variables, including age, gender, marital status, level of education, residence place, region, household economic level and health insurance status. IRR, incidence rate ratio; CI, confidence interval; PCE, Per capita household consumption expenditure.

**Table S4** Association of functional limitations with chronic conditions and socio-demographic factors (further adjusting for BMI, mental condition and physical activity)

| Variables | Limitation of ADL | | | | | | Limitation of IADL | | | | | |
| --- | --- | --- | --- | --- | --- | --- | --- | --- | --- | --- | --- | --- |
|  | OR^a^ | 95% CI | | OR^b^ | 95% CI | | OR^a^ | 95% CI | | OR^b^ | 95% CI | |
| No. of chronic disease (single) |  |  |  |  |  |  |  |  |  |  |  |  |
| Multimorbidity | 1.703 | 1.449 | 2.000 | 1.371 | 1.089 | 1.726 | 1.414 | 1.221 | 1.638 | 1.430 | 1.155 | 1.771 |
| Age (45-54) |  |  |  |  |  |  |  |  |  |  |  |  |
| 55-64 | 1.711 | 1.336 | 2.192 | 1.522 | 0.994 | 2.329 | 1.812 | 1.439 | 2.282 | 1.556 | 1.055 | 2.294 |
| 65-74 | 2.523 | 2.054 | 3.099 | 2.291 | 1.470 | 3.571 | 2.648 | 2.220 | 3.160 | 2.188 | 1.483 | 3.229 |
| 75 and above | 4.119 | 2.937 | 5.777 | 2.751 | 1.571 | 4.820 | 5.102 | 3.778 | 6.890 | 3.625 | 2.224 | 5.909 |
| Gender (male) |  |  |  |  |  |  |  |  |  |  |  |  |
| Female | 1.227 | 1.063 | 1.417 | 1.191 | 0.994 | 1.427 | 1.736 | 1.530 | 1.970 | 1.696 | 1.441 | 1.996 |
| Marital status (married) |  |  |  |  |  |  |  |  |  |  |  |  |
| Unmarried and other | 1.072 | 0.884 | 1.301 | 1.071 | 0.810 | 1.416 | 0.985 | 0.834 | 1.164 | 0.975 | 0.764 | 1.245 |
| Level of education (illiterate) |  |  |  |  |  |  |  |  |  |  |  |  |
| Primary school | 0.817 | 0.674 | 0.989 | 0.827 | 0.640 | 1.067 | 0.539 | 0.453 | 0.642 | 0.552 | 0.439 | 0.695 |
| Secondary school | 0.739 | 0.625 | 0.874 | 0.712 | 0.511 | 0.990 | 0.482 | 0.400 | 0.581 | 0.447 | 0.326 | 0.612 |
| College & above | 0.549 | 0.405 | 0.745 | 0.633 | 0.400 | 1.003 | 0.276 | 0.200 | 0.381 | 0.295 | 0.191 | 0.455 |
| Residence place (urban) |  |  |  |  |  |  |  |  |  |  |  |  |
| Rural | 1.383 | 1.159 | 1.651 | 1.429 | 1.097 | 1.860 | 1.371 | 1.155 | 1.626 | 1.316 | 1.028 | 1.685 |
| Region (east) |  |  |  |  |  |  |  |  |  |  |  |  |
| Central | 1.581 | 1.324 | 1.889 | 1.454 | 1.134 | 1.863 | 1.413 | 1.193 | 1.672 | 1.297 | 1.033 | 1.628 |
| West | 1.207 | 0.980 | 1.488 | 1.160 | 0.865 | 1.556 | 1.376 | 1.122 | 1.687 | 1.352 | 1.033 | 1.769 |
| PCE, quartile (Q1, the lowest) |  |  |  |  |  |  |  |  |  |  |  |  |
| Q2 | 1.017 | 0.829 | 1.247 | 0.984 | 0.730 | 1.325 | 0.895 | 0.747 | 1.072 | 1.015 | 0.772 | 1.335 |
| Q3 | 0.898 | 0.732 | 1.101 | 0.818 | 0.598 | 1.119 | 0.786 | 0.651 | 0.949 | 0.767 | 0.572 | 1.028 |
| Q4 (the highest) | 0.849 | 0.704 | 1.022 | 0.929 | 0.673 | 1.282 | 0.777 | 0.652 | 0.927 | 0.854 | 0.630 | 1.158 |
| Social health insurance (no) |  |  |  |  |  |  |  |  |  |  |  |  |
| Yes | 1.166 | 0.956 | 1.420 | 1.236 | 0.928 | 1.645 | 1.111 | 0.927 | 1.330 | 1.107 | 0.851 | 1.440 |
| BMI (≥18.5 to <25.0) |  |  |  |  |  |  |  |  |  |  |  |  |
| <18.5 | 0.899 | 0.695 | 1.163 | 0.746 | 0.524 | 1.061 | 1.367 | 1.080 | 1.731 | 1.604 | 1.157 | 2.225 |
| ≥25.0 to <30.0 | 1.317 | 1.139 | 1.523 | 1.299 | 1.009 | 1.672 | 1.070 | 0.936 | 1.222 | 1.043 | 0.828 | 1.314 |
| ≥30.0 | 1.881 | 1.460 | 2.423 | 1.764 | 1.192 | 2.610 | 1.117 | 0.852 | 1.463 | 1.001 | 0.659 | 1.519 |
| Depressive symptoms (no) |  |  |  |  |  |  |  |  |  |  |  |  |
| Yes | 3.310 | 2.928 | 3.741 | 2.886 | 2.342 | 3.557 | 2.843 | 2.526 | 3.199 | 2.660 | 2.210 | 3.202 |
| Physical activity (low level) |  |  |  |  |  |  |  |  |  |  |  |  |
| Moderate level | - | - | - | 0.664 | 0.508 | 0.870 | - | - | - | 0.603 | 0.476 | 0.762 |
| High level | - | - | - | 0.691 | 0.562 | 0.848 | - | - | - | 0.625 | 0.524 | 0.745 |

**Notes:** Logistic regression models were used to examine the association of functional limitations with chronic conditions. OR^a^, the odds ratios estimated by adjusting for age, gender, marital status, level of education, residence place, region, household economic level, health insurance status, BMI and depressive symptoms; OR^b^, adjusted for physical activity besides the covariates above. ADL, activities of daily living; IADL, instrumental activities of daily living; OR, odds ratio; CI, confidence interval; PCE, Per capita household consumption expenditure; BMI, Body mass index.

**Table S5** Association of work productivity loss with chronic conditions and socio-demographic factors (further adjusting for BMI, mental condition and physical activity)

| Variables | Limited work due to health problems | | | | | | Early retirement | | | | | |
| --- | --- | --- | --- | --- | --- | --- | --- | --- | --- | --- | --- | --- |
|  | OR^a^ | 95% CI | | OR^b^ | 95% CI | | OR^a^ | 95% CI | | OR^b^ | 95% CI | |
| No. of chronic disease (single) |  |  |  |  |  |  |  |  |  |  |  |  |
| Multimorbidity | 1.749 | 1.495 | 2.047 | 1.611 | 1.290 | 2.012 | 0.960 | 0.630 | 1.465 | 0.815 | 0.444 | 1.497 |
| Age (45-54) |  |  |  |  |  |  |  |  |  |  |  |  |
| 55-64 | 1.053 | 0.896 | 1.239 | 1.061 | 0.846 | 1.331 | 0.548 | 0.302 | 0.993 | 0.632 | 0.283 | 1.411 |
| 65-74 | 0.997 | 0.830 | 1.196 | 1.191 | 0.926 | 1.532 | 0.636 | 0.301 | 1.341 | 0.581 | 0.227 | 1.491 |
| 75 and above | 0.859 | 0.633 | 1.165 | 0.913 | 0.598 | 1.393 | 0.273 | 0.121 | 0.613 | 0.107 | 0.030 | 0.389 |
| Gender (male) |  |  |  |  |  |  |  |  |  |  |  |  |
| Female | 0.931 | 0.820 | 1.057 | 0.937 | 0.784 | 1.120 | 0.834 | 0.512 | 1.359 | 1.044 | 0.560 | 1.946 |
| Marital status (married) |  |  |  |  |  |  |  |  |  |  |  |  |
| Unmarried and other | 1.080 | 0.876 | 1.331 | 0.987 | 0.732 | 1.330 | 1.113 | 0.600 | 2.067 | 1.614 | 0.723 | 3.603 |
| Level of education (illiterate) |  |  |  |  |  |  |  |  |  |  |  |  |
| Primary school | 1.109 | 0.944 | 1.302 | 1.144 | 0.917 | 1.427 | 1.191 | 0.634 | 2.237 | 1.206 | 0.526 | 2.763 |
| Secondary school | 0.915 | 0.767 | 1.091 | 0.961 | 0.756 | 1.222 | 2.021 | 1.057 | 3.865 | 1.646 | 0.712 | 3.806 |
| College & above | 0.691 | 0.513 | 0.929 | 0.549 | 0.365 | 0.825 | 2.049 | 1.159 | 3.621 | 1.538 | 0.708 | 3.341 |
| Residence place (urban) |  |  |  |  |  |  |  |  |  |  |  |  |
| Rural | 1.547 | 1.316 | 1.818 | 1.403 | 1.151 | 1.711 | 1.094 | 0.692 | 1.728 | 1.400 | 0.697 | 2.812 |
| Region (east) |  |  |  |  |  |  |  |  |  |  |  |  |
| Central | 1.156 | 0.977 | 1.368 | 1.034 | 0.846 | 1.264 | 1.222 | 0.854 | 1.749 | 1.434 | 0.826 | 2.490 |
| West | 0.964 | 0.793 | 1.173 | 0.915 | 0.707 | 1.184 | 1.838 | 1.067 | 3.169 | 2.149 | 0.970 | 4.762 |
| PCE, quartile (Q1, the lowest) |  |  |  |  |  |  |  |  |  |  |  |  |
| Q2 | 1.007 | 0.848 | 1.195 | 1.063 | 0.847 | 1.334 | 1.054 | 0.523 | 2.125 | 1.381 | 0.446 | 4.276 |
| Q3 | 0.828 | 0.691 | 0.993 | 0.753 | 0.591 | 0.960 | 0.623 | 0.318 | 1.223 | 0.632 | 0.228 | 1.752 |
| Q4 (the highest) | 0.688 | 0.565 | 0.838 | 0.806 | 0.627 | 1.038 | 1.101 | 0.577 | 2.100 | 1.045 | 0.393 | 2.779 |
| Social health insurance (no) |  |  |  |  |  |  |  |  |  |  |  |  |
| Yes | 1.215 | 0.997 | 1.480 | 1.182 | 0.906 | 1.542 | 1.133 | 0.630 | 2.036 | 1.222 | 0.511 | 2.922 |
| BMI (≥18.5 to <25.0) |  |  |  |  |  |  |  |  |  |  |  |  |
| <18.5 | 1.302 | 1.019 | 1.664 | 1.363 | 0.962 | 1.929 | 0.407 | 0.120 | 1.379 | 0.267 | 0.057 | 1.252 |
| ≥25.0 to <30.0 | 1.004 | 0.874 | 1.153 | 1.030 | 0.852 | 1.246 | 1.179 | 0.770 | 1.806 | 1.200 | 0.702 | 2.050 |
| ≥30.0 | 1.326 | 1.031 | 1.704 | 1.119 | 0.796 | 1.574 | 1.100 | 0.552 | 2.192 | 0.419 | 0.114 | 1.540 |
| Depressive symptoms (no) |  |  |  |  |  |  |  |  |  |  |  |  |
| Yes | 3.140 | 2.754 | 3.579 | 3.229 | 2.690 | 3.876 | 1.759 | 0.941 | 3.288 | 1.314 | 0.735 | 2.350 |
| Physical activity (low level) |  |  |  |  |  |  |  |  |  |  |  |  |
| Moderate level | - | - | - | 0.803 | 0.646 | 0.999 | - | - | - | 0.817 | 0.493 | 1.354 |
| High level | - | - | - | 0.733 | 0.603 | 0.890 | - | - | - | 0.599 | 0.294 | 1.221 |

**Notes:** Logistic regression models were used in this table. OR^a^, the odds ratios estimated by adjusting for age, gender, marital status, level of education, residence place, region, household economic level, health insurance status, BMI and depressive symptoms; OR^b^, adjusted for physical activity besides the covariates above. ADL, activities of daily living; IADL, instrumental activities of daily living; OR, odds ratio; CI, confidence interval; PCE, Per capita household consumption expenditure; BMI, Body mass index.

**Table S6** Association of days of sick leave with chronic conditions and socio-demographic factors (further adjusting for BMI, mental condition and physical activity)

| Variables | Sick leave at agricultural work | | | | | | Sick leave at employed non-agricultural work | | | | | |
| --- | --- | --- | --- | --- | --- | --- | --- | --- | --- | --- | --- | --- |
|  | IRR^a^ | 95% CI | | IRR^b^ | 95% CI | | IRR^a^ | 95% CI | | IRR^b^ | 95% CI | |
| No. of chronic disease (single) |  |  |  |  |  |  |  |  |  |  |  |  |
| Multimorbidity | 1.646 | 1.356 | 1.998 | 1.558 | 1.185 | 2.048 | 2.644 | 1.284 | 5.443 | 1.827 | 0.809 | 4.130 |
| Age (45-54) |  |  |  |  |  |  |  |  |  |  |  |  |
| 55-64 | 1.140 | 0.928 | 1.400 | 1.244 | 0.958 | 1.615 | 0.653 | 0.342 | 1.248 | 0.506 | 0.177 | 1.444 |
| 65-74 | 1.386 | 1.096 | 1.752 | 1.533 | 1.144 | 2.055 | - | - | - | - | - | - |
| 75 and above | 1.464 | 0.962 | 2.228 | 0.801 | 0.500 | 1.282 | - | - | - | - | - | - |
| Gender (male) |  |  |  |  |  |  |  |  |  |  |  |  |
| Female | 1.055 | 0.893 | 1.247 | 1.027 | 0.816 | 1.292 | 0.627 | 0.335 | 1.176 | 0.486 | 0.217 | 1.091 |
| Marital status (married) |  |  |  |  |  |  |  |  |  |  |  |  |
| Unmarried and other | 1.031 | 0.713 | 1.490 | 1.298 | 0.785 | 2.146 | 3.138 | 0.672 | 14.641 | 0.143 | 0.041 | 0.499 |
| Level of education (illiterate) |  |  |  |  |  |  |  |  |  |  |  |  |
| Primary school | 0.976 | 0.792 | 1.202 | 0.869 | 0.660 | 1.143 | 1.719 | 0.675 | 4.380 | 1.081 | 0.410 | 2.847 |
| Secondary school | 0.703 | 0.566 | 0.873 | 0.746 | 0.576 | 0.965 | 2.415 | 1.247 | 4.677 | 3.469 | 1.591 | 7.567 |
| College & above | 0.969 | 0.604 | 1.554 | 0.711 | 0.371 | 1.363 | 1.370 | 0.605 | 3.098 | 1.199 | 0.437 | 3.292 |
| Residence place (urban) |  |  |  |  |  |  |  |  |  |  |  |  |
| Rural | 1.543 | 1.232 | 1.933 | 1.592 | 1.222 | 2.074 | 1.064 | 0.612 | 1.847 | 0.682 | 0.315 | 1.476 |
| Region (east) |  |  |  |  |  |  |  |  |  |  |  |  |
| Central | 1.025 | 0.819 | 1.284 | 0.936 | 0.694 | 1.263 | 0.944 | 0.503 | 1.771 | 0.664 | 0.299 | 1.478 |
| West | 1.231 | 0.983 | 1.542 | 1.353 | 1.009 | 1.814 | 0.814 | 0.402 | 1.648 | 1.453 | 0.473 | 4.458 |
| PCE, quartile (Q1, the lowest) |  |  |  |  |  |  |  |  |  |  |  |  |
| Q2 | 0.848 | 0.676 | 1.064 | 0.825 | 0.621 | 1.094 | 1.985 | 0.606 | 6.507 | 1.423 | 0.316 | 6.414 |
| Q3 | 1.164 | 0.938 | 1.445 | 1.167 | 0.872 | 1.562 | 1.012 | 0.388 | 2.641 | 1.726 | 0.370 | 8.040 |
| Q4 (the highest) | 1.058 | 0.856 | 1.307 | 1.127 | 0.857 | 1.483 | 2.796 | 1.130 | 6.915 | 4.647 | 1.026 | 21.048 |
| Social health insurance (no) |  |  |  |  |  |  |  |  |  |  |  |  |
| Yes | 1.054 | 0.790 | 1.405 | 1.311 | 0.892 | 1.927 | 1.423 | 0.619 | 3.272 | 0.561 | 0.224 | 1.403 |
| BMI (≥18.5 to <25.0) |  |  |  |  |  |  |  |  |  |  |  |  |
| <18.5 | 1.090 | 0.815 | 1.457 | 1.458 | 0.983 | 2.164 | 2.288 | 0.451 | 11.615 | 1.605 | 0.129 | 19.959 |
| ≥25.0 to <30.0 | 1.205 | 1.004 | 1.447 | 1.273 | 1.021 | 1.587 | 0.887 | 0.493 | 1.597 | 1.377 | 0.662 | 2.863 |
| ≥30.0 | 0.965 | 0.663 | 1.402 | 0.920 | 0.590 | 1.437 | 1.656 | 0.337 | 8.135 | 0.024 | 0.004 | 0.151 |
| Depressive symptoms (no) |  |  |  |  |  |  |  |  |  |  |  |  |
| Yes | 2.318 | 1.969 | 2.727 | 2.070 | 1.659 | 2.581 | 3.422 | 1.706 | 6.866 | 6.114 | 2.399 | 15.578 |
| Physical activity (low level) |  |  |  |  |  |  |  |  |  |  |  |  |
| Moderate level | - | - | - | 0.694 | 0.505 | 0.955 | - | - | - | 1.239 | 0.427 | 3.600 |
| High level | - | - | - | 0.804 | 0.610 | 1.060 | - | - | - | 1.682 | 0.599 | 4.724 |

**Notes:** Negative binomial regression models were used to assess the association between days of sick leave with chronic conditions. IRR^a^, the incidence rate ratios estimated by adjusting for age, gender, marital status, level of education, residence place, region, household economic level, health insurance status, BMI and depressive symptoms; IRR^b^, adjusted for physical activity besides the covariates above. ADL, activities of daily living; IADL, instrumental activities of daily living; OR, odds ratio; CI, confidence interval; PCE, Per capita household consumption expenditure; BMI, Body mass index.
